# Effects of Agricultural Management of Spent Mushroom Waste on Phytotoxicity and Microbiological Transformations of C, P, and S in Soil and Their Consequences for the Greenhouse Effect

**DOI:** 10.3390/ijerph191912915

**Published:** 2022-10-09

**Authors:** Edyta Kwiatkowska, Jolanta Joniec

**Affiliations:** Department of Environmental Microbiology, Faculty of Agrobioengineering, University of Life Sciences in Lublin, Leszczyńskiego 7, 20-069 Lublin, Poland

**Keywords:** spent mushroom substrate, manure, phytotoxicity, soil respiration, greenhouse effect, dehydrogenases, enzymatic activity, *Lepidium sativum* L., waste, soil microorganisms

## Abstract

The huge volumes of currently generated agricultural waste pose a challenge to the economy of the 21st century. One of the directions for their reuse may be as fertilizer. Spent mushroom substrate (SMS) could become an alternative to manure (M). A three-year field experiment was carried out, in which the purpose was to test and compare the effect of SMS alone, as well as in multiple variants with mineral fertilization, and in manure with a variety of soil quality indices—such as enzymatic activity, soil phytotoxicity, and greenhouse gas emissions, i.e., CO_2_. The use of SMS resulted in significant stimulation of respiratory and dehydrogenase activity. Inhibition of acid phosphatase and arylsulfatase activity via SMS was recorded. SMS showed varying effects on soil phytotoxicity, dependent on time. A positive effect was noted for the growth index (GI), while inhibition of root growth was observed in the first two years of the experiment. The effect of M on soil respiratory and dehydrogenase activity was significantly weaker compared to SMS. Therefore, M is a safer fertilizer as it does not cause a significant persistent increase in CO_2_ emissions. Changes in the phytotoxicity parameters of the soil fertilized with manure, however, showed a similar trend as in the soil fertilized with SMS.

## 1. Introduction

The intensification of human economic and livelihood activities is associated with the generation of huge amounts of various types of waste [1]. Therefore, the modern economy is increasingly open to production based on technologies that allow the integration of the broadly understood waste back into the production cycle. Among the many directions for their reuse, fertilizer application is particularly important. Agricultural wastes generated in rural areas as a result of crop processing and agricultural activities show a particularly high fertilizing potential. One such waste of organic origin is spent mushroom substrate (SMS) (*Agaricus bisporus* L.) [2].

According to the Food and Agriculture Organization Corporate Statistical Database, the global production of mushrooms and truffles in 2020 was 42,792,893 tons compared, for example, to only 8,781,004 tons in 2000, i.e., 20% of the total current production. Globally, China is, by far, the main producer of mushrooms and truffles (40,004,574 tons in 2020), while Europe (1,270,241 tons in 2020) is led mainly by the Netherlands (260,000 tons in 2020), Poland (182,900 tons in 2020), and Spain (166,010 tons in 2020) [3]. Such intensive global production results in the generation of large quantities of spent mushroom substrate, estimated at approximately 60 million tons per year [4,5]. The efficient use and disposal of such a large volume of annually generated material is, therefore, a major challenge for the modern economy.

Due to its composition (mainly high organic matter content), poorly stored spent mushroom substrate can pose environmental hazards through the development of pathogenic microflora and the spread of fungal diseases, uncontrolled waste biodegradation by microorganisms, and the consequent emission of greenhouse gases into the atmosphere; this is as well as the leaching of nutrients into surface and groundwater [5,6]. 

Due to growing environmental concerns, the proper disposal and handling of excess SMS accumulation are essential. Current research has clearly indicated that, due to its high fertilizing value, agricultural application is the most efficient method for SMS recycling [7,8,9,10]. SMS is a valuable source of organic matter and nutrients that is readily available to plants [8,11,12]. It is important to note that the composition of SMS varies greatly depending on location, type of mushroom grown, and other factors [5]. It also improves a number of soil properties, including structure, pH, and water-holding capacity [13,14]. Additionally, this method of management indirectly solves the problem of other wastes, i.e., those previously used to compose mushroom substrate, e.g., straw; poultry and cattle manure, waste gypsum from electrostatic precipitators, phosphogypsum and CaCO_3_ [2,5]. In addition, spent mushroom substrate can be composted with the addition of other wastes, i.e., liquid manure or sewage sludge, which also allows for the recycling of these additional wastes [15,16]. Considering the wide variety and variability of individual spent mushroom substrates, it is advisable to study their composition and possibly balance the components by supplementing them with mineral fertilization.

It is important to carry out soil toxicity tests due to the possibility of toxic compound formation, which arises as a result of the microbiological transformation of waste organic matter. In order to monitor the soil environment in this respect, it is recommended to use biotests, e.g., a phytotest using *Lepidium sativum* L. [17,18]. *L. sativum* L. has been repeatedly used as a bio-indicator to determine the effects of various chemical compounds, including those of waste origin, on plant germination and growth [19,20,21,22,23]. 

When selecting the method of managing organic waste, including waste generated in agriculture, one should take into account the possibility of greenhouse gas (GHG) emissions as a result of the transformation of carbon and nitrogen matter. Agriculture is the main sector contributing to their emissions, estimated at between 10% and 20% of the total anthropogenic GHG emissions [24]. Both fertilizers and waste, especially organic waste, contain large amounts of organic carbon, whose resources in farmland play a key role in sustainable agriculture. It is the organic matter, the main source of which can be SMS, that influences the rate of mineralization, accumulation, or emission of carbon from the soil and the complex interactions between biological and physico-chemical soil processes and environmental conditions [25]. Soil carbon sequestration, i.e. increasing the amount of this element in the soil, stored as organic matter, can improve soil quality and reduce the contribution of agriculture to CO_2_ emissions [26,27]. However, simply adding organic matter to the soil will not solve the problem. In addition, it is also important to assess the impact of this application on soil processes and microbial activity. As approximately 90% of CO_2_ emitted from the soil is of microbial origin, it is, therefore, the main component in the global carbon cycle, emitting about ten times more CO_2_ per year into the atmosphere than burning fossil fuels [28,29]. Therefore, changes in the activity of respiratory processes may indicate ecological disturbances and also a large contribution of microorganisms to soil metabolism and global warming. An indirect indicator of the total number and activity of microorganisms in the soil is respiratory activity, which can be a marker of changes occurring in this environment [30]. CO_2_ emitted from the soil is the final product of mineralization and the oxidation of organic substances by soil microorganisms, but also the result of plant respiratory processes and the decomposition of organic compounds brought into the soil with roots [31]. Therefore, respiratory activity has been recognized by many other authors as a good determinant of the rate of organic matter decomposition or microbial biomass [21,32,33,34,35].

Soil enzymes also play a significant role in ecosystem processes, participating in multiple reactions that are an integral part of various biogeochemical cycles [36]. They regulate, among others, the decomposition of organic matter and determine the availability of nutrients in the soil; therefore, they are critical for the carbon cycle in ecosystems [37,38]. Given that microorganisms contribute significantly to organic matter cycling and long-term soil carbon stabilization, it is thus necessary to monitor the impact of climate change (this includes, in particular, the greenhouse effect), on microbial communities and soil carbon cycling rates. Enzyme activity reflects the metabolic requirements of the microbial community and may therefore be an important indicator of microbial function in response to climate change [39,40]. Both acid phosphatase and arylsulfatase have been used multiple times to assess the condition of soil environments, including those fertilized with various types of organic waste [21,34,41,42,43]. 

A number of agricultural wastes, including spent mushroom substrate, have significant fertilizing potential. A multi-year field study was conducted as part of a series [44] to investigate and compare the effects of spent mushroom waste and manure on soil quality indicators, such as biochemical and enzymatic activity related to microbial transformations of C, P, and S, as well as soil phytotoxicity. Pertaining to this research, the authors posed the following hypotheses: (1) spent mushroom waste is a good fertilizer alternative to manure and can be applied annually; (2) spent mushroom waste has no phytotoxic effects on the initial stages of plant growth, i.e., germination, root growth, and sprout weight; (3) agricultural management of SMS does not contribute to the greenhouse effect. In view of the above assumptions, the authors assumed that the obtained results would allow for better management of agricultural waste, including spent mushroom waste, in a manner that ensures an increase in soil fertility in accordance with the principle of sustainable development. The presented research may be helpful in achieving the United Nations’ Sustainable Development Goals (SDGs), such as climate action, responsible consumption and production, and the elimination of poverty and hunger [45,46].

## 2. Materials and Methods

### 2.1. Site and Experimental Setup and Soil Sampling 

**Figure 1:**
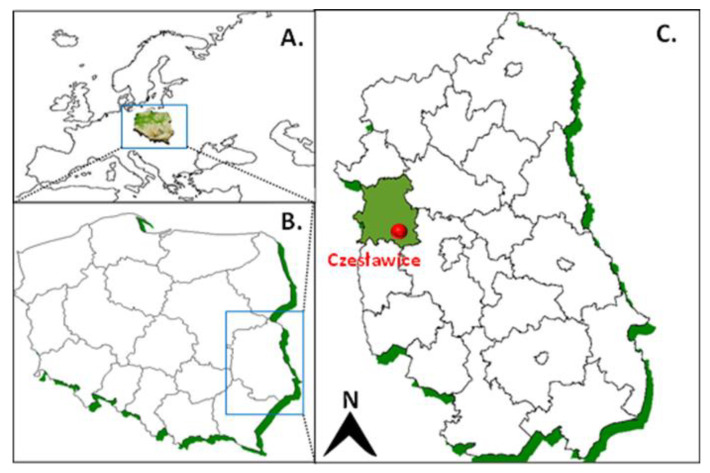
Location of the research area: (**A**) location of Poland against the background of Europe; (**B**) location of the Lublin region in Poland; and (**C**) location of the “Czesławice” farm in the Lublin region.

The experiment with the use of spent mushroom substrate and manure was carried out at the Czesławice Experimental Station (Lublin region, Poland, 51°18′26″ N, 22°16′1″ E) (Figure 1) in a randomized block design.

Experimental plots were located on a lessive soil belonging to the second quality class [47,48]. Soil grain size composition was as follows: fraction 1.0–0.1 mm—medium sand (4%); fraction 0.1–0.02 mm—fine sand—coarse dust (52%); fraction 0.02–0.002 mm—fine dust (35%); and fraction <0.002 mm—colloidal clay (9%). The plots were established in triplicate (the area of a single plot was 3 m^2^) and fertilized for three years (in the fall) with single doses (20 t ha^−1^) of spent mushroom medium SMS (moisture 67%) and composted cattle manure M (moisture 77%). The spent mushroom medium was composed on the basis of winter wheat straw, peat, and chicken manure. It did not contain any mineral additives, as it was intended for organic farming. Supplemental mineral fertilization with nitrogen (N), phosphorus (P), and potassium (K) was also applied to the sites with this substrate. This was due to the initial abundance of assimilable nutrients in the soil and from the hypothesized rapid release of nutrients from this waste, and thus the short-term fertilizing effect of the spent mushroom substrate alone (without NPK fertilization). Therefore, nitrogen was introduced in the form of ammonium nitrate at doses of N1—50 kg ha^−1^ and N2—100 kg ha^−1^, phosphorus in the form of granular triple superphosphate at doses of P1—30 kg ha^−1^ and P2—60 kg ha^−1^, and potassium as potassium sulfate at K1—70 kg ha^−1^ and K2—140 kg ha^−1^. Soil without fertilizer constituted the control object. Italian ryegrass (*Lolium multiflorum* Lam.) was used as the test plant. The characteristics of the spent mushroom substrate and manure are presented in Table 1.

Experimental scheme:

1. Soil without fertilizer (control object) (C);
2. Soil + spent mushroom substrate (SMS);
3. Soil + spent mushroom substrate + N1P1K1 (SMS + N1P1K1);
4. Soil + spent mushroom substrate + N2P2K2 (SMS + N2P2K2);
5. Soil + cattle manure (M).

Research was carried out from 2018 to 2020. Soil material was collected with a gouging drill, from the 0–25 cm layer, from ten randomly selected sites within each test plot at two time points, i.e., in the spring (June) and fall (September). The average soil sample from each plot (about 4 kg) consisted of a mixture of 10 soil cores, each 4 cm in diameter. The collected samples were sifted through a 2 mm sieve and stored in plastic bags at 4 °C.

### 2.2. Meteorological Conditions

**Figure 2:**
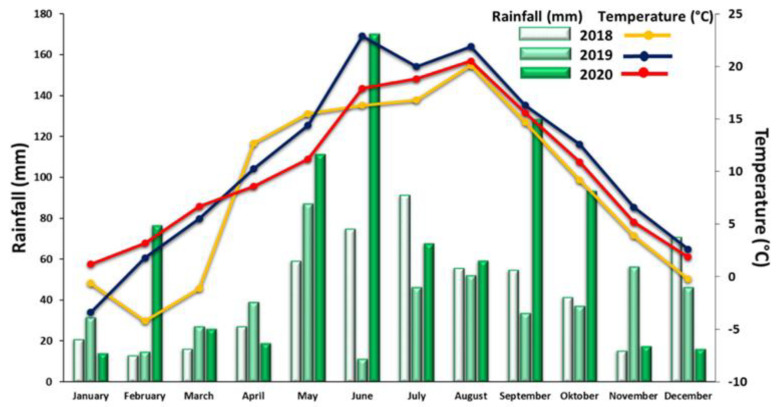
Average monthly temperatures and monthly rainfall totals in the experimental area during the research period.

Weather conditions were recorded by the Meteorological Station in Czesławice, located ~800 m from the field experiment. The total precipitation in 2018, 2019, and 2020 was 539.3, 481.8, and 799.7 mm, respectively, while the average annual air temperature was 8.6, 11.0, and 10.1 °C, respectively. Analyzing the weather conditions in the months of soil sampling—i.e., June and September—it was found that monthly precipitation varied considerably and amounted to 74.8 and 54.7 in 2018, 11.2 and 33.5 in 2019, and 170.3 and 128.5 in 2020. The highest temperature during the entire study period was observed in June 2019 (22.9 °C), while values of 16.3, 17.9, 14.7, 16.3, and 15.6 were recorded at the remaining time points—June 2018 and 2020, and September 2018, 2019, and 2020, respectively (Figure 2).

### 2.3. Biochemical and Enzymatic Analyses

Respiratory activity was determined using the method of Rühling and Tyler [49]. Soil samples (20 g) with 1% glucose addition were incubated for 24 h in the presence of 0.2 M NaOH solution. After incubation, the excess unbound sodium hydroxide was titrated with 0.1 M HCl in the presence of BaCl_2_ and phenolphthalein. 

Thalmann’s [50] method was used to determine dehydrogenase activity. Further, soil samples (5 g) with 2,3,5–triphenyltetrazolium chloride addition as the substrate were incubated in 0.1 M tris(hydroxymethyl)aminomethane buffer (Tris–HCl pH 7.4) for 48 h at 30 °C. Enzymatic activity was determined colorimetrically (λ = 485 nm) by measuring the extinction of the TPF (triphenylformazan) produced. 

The method of Tabatabai and Bremner [51] was used to determine acid phosphatase activity. Soil samples (1 g) with p-nitrophenyl disodium phosphate (PNPNa) as a substrate were incubated for one hour at 37 °C in a modified universal buffer (pH 6.5). For arylsulfatase, soil samples (1 g) were incubated for 1 h at 37 °C in the presence of p-nitrophenol sulfate (PNS) in a modified universal buffer (pH 5.8) [52]. The activity of both enzymes was determined spectrophotometrically at 400 nm and expressed as para-nitrophenol-mg PNP kg^−^^1^ dm soil h^−^^1^. 

All analyses were carried out in triplicate, and activities were calculated based on dry soil weight.

### 2.4. Phytotoxicity

As part of the soil phytotoxicity evaluation, two phytotests were performed using garden cress (*Lepidium sativum*) as a test plant.

The test of Masciandaro et al. [53] was used for the purposes of determining the effect of the overall conditions in the soil on the development of *L. sativum*, following the application of the tested variants of organic fertilization. For this purpose, 100 seeds of *L. sativum* were sown (in triplicate) on 50-gram weights of fresh soil placed in Petri dishes (moisture content—60%WHC). Incubation was carried out for four days at 22 °C, maintaining a constant moisture level. Subsequently, the number of germinated seeds was counted and their weight was determined. The growth index (GI) was calculated based on these parameters, according to the formula of Masciandaro et al. [53]:

GI%=P (TC)

*P*—mean % of germinated seeds in the reclaimed soil relative to the value for the control soil; *T*—mean weight of fresh *L. sativum* sprouts in the reclaimed soil; *C*—mean weight of fresh *L. sativum* sprouts in the control soil.

The second test analyzed the effect of potentially toxic substances dissolved in soil solution on the sprouting and growth of *L. sativum* roots after 2 and 4 days. For this purpose, fresh soil weights (20 g) (moisture content—60%WHC) were placed on Petri dishes covered with sterile disks of blotting paper in six replicates. Following this, 90 *L. sativum* seeds were placed on 3 plates, and 10 seeds on the remaining 3 plates. Incubation was carried out at 22 °C. The number of germinated seeds on all plates was counted after two days. The length of sprout roots was also measured after two and four days on plates containing ten seeds each.

### 2.5. Chemical Analyses 

Chemical analyses complemented biochemical, enzymatic, and phytotoxicity tests (Table 1 and Table 2). The pH was determined from the soil extract in KCl (10 g of soil in 25 mL of KCl) using an electrometric method. Organic carbon (TOC) was determined by IR spectrometry. The Kjeldahl method was used to determine total nitrogen (TN), and total phosphorus (TP) was determined by spectrophotometry. Flame atomic absorption spectrometry (FAAS) was used to determine calcium, potassium, and magnesium. All of the above methods were applied to soil, as well as spent mushroom substrate and manure samples. In addition, heavy metals in the spent mushroom substrate were determined using atomic absorption spectroscopy (AAS).

### 2.6. Statistical Analysis 

Descriptive statistics involved calculating the arithmetic means of three replicates obtained for a given sample, along with the standard deviation. The results were presented in the form of bar graphs. Statistical evaluation of result variability was carried out using a two-factor analysis of variance, where each year was analyzed separately. The basic ANOVA assumptions, including normality of the dataset and homogeneity of variance, were checked with the Shapiro–Wilk and Levene tests. The significance of differences between means was verified using Tukey’s post hoc test. Significance was assumed at α=0.05. The relationships between the analyzed biochemical, enzymatic, phytotoxic, and physicochemical parameters and environmental conditions were analyzed via principal component analysis (PCA). These relationships were also analyzed at the level of experimental combinations using Pearson correlations at three levels of significance: *p* < 0.001, *p* < 0.01, and *p* < 0.05; in addition, the results were presented as heat maps. All statistical analyses were performed using the Statistica 13.1 package (TIBCO Software Inc.; Palo Alto, CA, USA).

## 3. Results 

**Figure 3:**
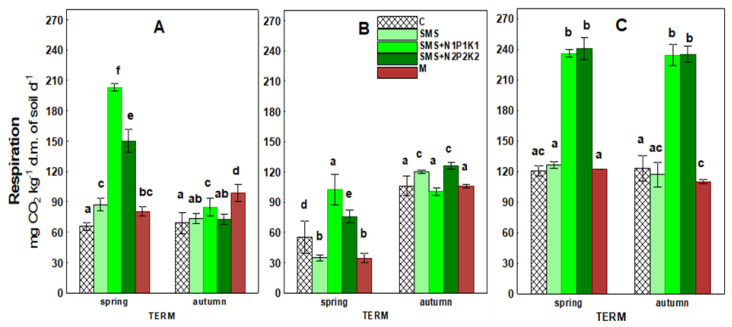
Respiratory activity in control soil and soil under different treatment strategies. (**A**) 1st year; (**B**) 2nd year; and (**C**) 3rd year. C—control soil; SMS—soil + spent mushroom substrate; SMS + N1P1K1—soil + spent mushroom substrate + mineral fertilization N1P1K1; SMS + N2P2K2—soil + spent mushroom substrate + mineral fertilization N2P2K2; and M—soil + manure. The vertical lines indicate the standard deviation. Different letters above the columns indicate significant differences at *p* < 0.05, and each year was analyzed independent of each other.

The data presented in Figure 3 and Table 3 show that the application of spent mushroom substrate and manure significantly affected soil respiration. During the three years of the experiment, this activity was stimulated in the spent mushroom substrate sites (SMS, SMS + N1P1K1, and SMS + N2P2K2), and its intensity varied from site to site and changed with time. Respiration reached the highest values in the third year in the sites with NPK fertilization—i.e., SMS + N1P1K1 and SMS + N2P2K2 (234.08–240.72 mg)—while the lowest values were recorded in the second year in the site with spent mushroom substrate alone, i.e., SMS (34.78 mg). The introduction of waste into the soil separately and in combination with NPK at both doses resulted in the stimulation of respiration, which over time was limited to the sites where SMS was introduced together with NPK (SMS + N1P1K1 and SMS + N2P2K2). The highest stimulation of this parameter was recorded at these sites in the spring of the first year and in the spring and fall of the third year. In contrast, the impact of SMS alone was not as directional over time. In the second year, a decrease and then a subsequent increase in respiration were recorded under its influence, and in the third year, its effect disappeared.

The effect of manure on respiration was significantly less apparent than that of the spent mushroom substrate. Stimulation of this process was recorded in the manure sites only in the first year and occurred more strongly in the fall, where its value was 98.74 mg compared to 68.95 mg in the control (C). In subsequent years, there was a loss of stimulation and even a decrease in respiratory activity.

**Figure 4:**
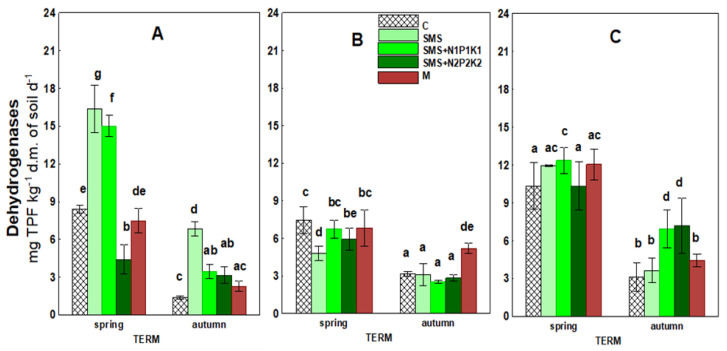
Activity of dehydrogenases in control soil and soil under different treatment strategies. (**A**)—1st year; (**B**)—2nd year; (**C**)—3rd year. C—control soil; SMS—soil + spent mushroom substrate; SMS + N1P1K1—soil + spent mushroom substrate + mineral fertilization N1P1K1; SMS + N2P2K2—soil + spent mushroom substrate + mineral fertilization N2P2K2; M—soil + manure. The vertical lines indicate the standard deviation. Different letters above the columns indicate significant differences at *p* < 0.05, and each year was analyzed independent of each other.

Figure 4 and Table 3 show data concerning dehydrogenase activity. The introduction of a spent mushroom substrate in various combinations and manure into the soil caused significant changes in the activity of these enzymes. As with respiration, this parameter was also generally subject to stimulation under the influence of the spent mushroom substrate. This effect also lasted the longest at sites where spent mushroom substrate was introduced in combination with NPK (SMS + N1P1K1, SMS + N2P2K2). The dynamics of changes over time were similar to that recorded for respiration. The highest stimulation was recorded in the first year of the study, where the value of enzymatic activity in the spring at the SMS only site was 16.37 mg, in the fall 6.84 mg, while only 8.41 mg and 1.38 mg in the control (C), respectively. The positive effect of SMS disappeared in the second year of the study, and there was even a decrease recorded in dehydrogenase activity in the spring. However, in the third year of SMS application, its stimulating effect occurred again in the sites with mineral fertilization (SMS + N1P1K1 and SMS + N2P2K2).

Manure, as in the case of respiration, had a significantly weaker effect on dehydrogenase activity than the spent mushroom substrate. Its significant impact was recorded only in autumn in the second year in the form of an increase in this parameter. The enzyme activity value in the manure site (M) was 5.20 mg at this time point, while in control (C), it was 3.18 mg.

**Figure 5:**
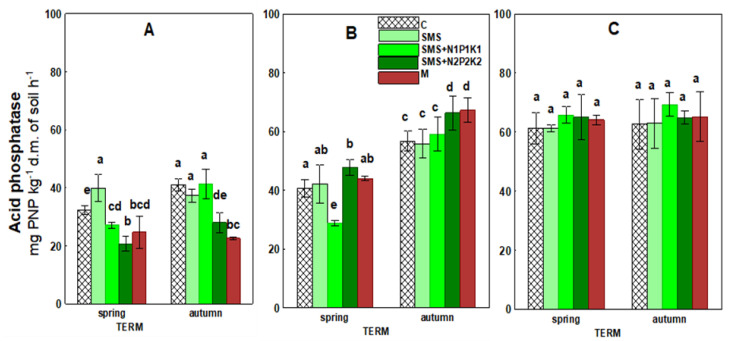
Acid phosphatase activity in control soil and soil under different treatment strategies. (**A**)—1st year; (**B**)—2nd year; (**C**)—3rd year. C—control soil; SMS—soil + spent mushroom substrate; SMS + N1P1K1—soil + spent mushroom substrate + mineral fertilization N1P1K1; SMS + N2P2K2—soil + spent mushroom substrate + mineral fertilization N2P2K2; M—soil + manure. The vertical lines indicate the standard deviation. Different letters above the columns indicate significant differences at *p* < 0.05, and each year was analyzed independent of each other.

Figure 5 and Table 3 present data regarding acid phosphatase activity. The results showed that the impact of SMS was not directional and exhibited varying intensity throughout the study period. During the first two years of the experiment, it caused a decrease, an increase, or no significant effect on the discussed enzymatic activity at individual time points, depending on the variant in which the spent mushroom substrate was applied. The use of SMS alone had an effect only in the first time point in the form of phosphatase activity stimulation. The introduction of SMS in combination with NPK fertilization (SMS + N1P1K1 and SMS + N2P2K2), on the other hand, caused a decrease in activity at this time point. This effect apparently occurred in the fall in the facility with a higher NPK dose (SMS + N2P2K2). The activity at this site was 27.99 mg, while it was 41.00 mg in control (C). The negative effect of SMS at this site disappeared over time, and stimulation of phosphatase activity was already observed in the second year. The value of this parameter at this site (SMS+N2P2K2) was 66.36 mg, while it was 56.76 mg in the control (C). The impact of the waste at all sites completely disappeared in the third year of the study.

The effect of manure was also not directional. In the first year, there was a decrease in phosphatase activity at this site (M) and at both time points. In the following years, a significant impact was recorded only in the second year in the fall in the form of stimulation. It should be noted that phosphatase activity reached the highest value of 67.24 mg in this time point compared to 56.76 mg in the control.

**Figure 6:**
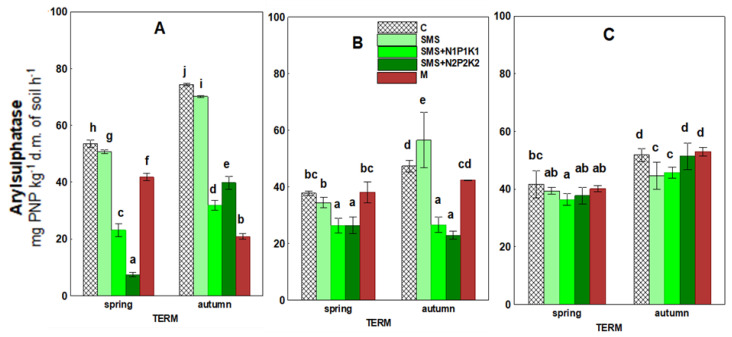
Arylsulfatase activity in control soil and soil under different treatment strategies. (**A**)—1st year; (**B**)—2nd year; (**C**)—3rd year. C—control soil; SMS—soil + spent mushroom substrate; SMS + N1P1K1—soil + spent mushroom substrate + mineral fertilization N1P1K1; SMS + N2P2K2—soil + spent mushroom substrate + mineral fertilization N2P2K2; M—soil + manure. The vertical lines indicate the standard deviation. Different letters above the columns indicate significant differences at *p* < 0.05, and each year was analyzed independent of each other.

The effect of spent mushroom substrate and manure on arylsulfatase activity, as opposed to phosphatase, was directional (Figure 6, Table 3). The activity of arylsulfatase was subject to significant inhibition persisting in the spent mushroom substrate with varying intensity throughout the study period. In the first year, spent mushroom substrate, introduced both separately and together with both NPK doses (SMS, SMS + N1P1K1, and SMS + N2P2K2), caused a decrease in arylsulfatase activity. It should be noted that inhibition was the strongest during this year, and the value of this parameter in the SMS and N1P1K1 site was only 7.38 mg, while in the control (C) it was 56.56 mg. The negative effect of waste also persisted in the second year in the sites with NPK fertilization (SMS + N1P1K1 and SMS + N2P2K2) and in the third year in the site with SMS and N1P1K1. The only positive effect that was noted for spent mushroom substrate (SMS) was in the fall in the second year of the study.

The application of manure induced significant changes in arylsulfatase activity, but only in the first year of the study. There was a decrease in this parameter in both spring and fall. In the fall, the activity of this enzyme was the lowest of all the time points for this site (M) and was 20.81 mg compared to 74.33 mg in the control.

**Figure 7:**
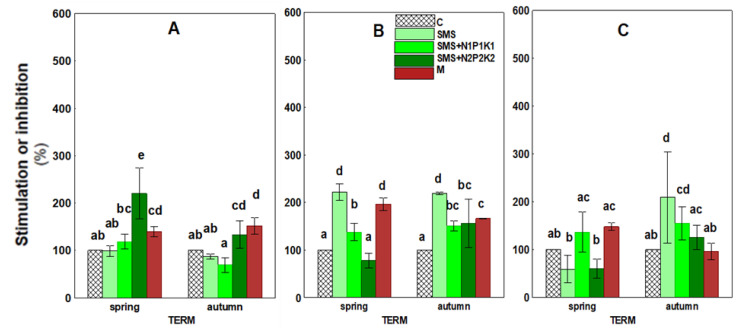
Growth index *Lepidium sativum* in soil under different treatment strategies. (**A**)—1st year; (**B**)—2nd year; (**C**)—3rd year. C—control soil; SMS—soil + spent mushroom substrate; SMS + N1P1K1—soil + spent mushroom substrate + mineral fertilization N1P1K1; SMS + N2P2K2—soil + spent mushroom substrate + mineral fertilization N2P2K2; M—soil + manure. The vertical lines indicate the standard deviation. Different letters above the columns indicate significant differences at *p* < 0.05, and each year was analyzed independent of each other.

Figure 7 and Table 3 present the growth index (GI) data of the test plant. Data analysis showed that the introduction of SMS and manure into the soil resulted in an increase in this parameter. Initially, this effect became apparent only in sites where SMS was applied in combination with N2P2K2 fertilization. 

In time, i.e., in the second year, the stimulation of this parameter intensified and, in the fall, it was already visible in all sites with waste. The highest stimulation that was recorded was for SMS alone, and the values recorded were 121.67% and 118.17%. In the third year of the study, the recorded stimulation weakened and was evident in fewer sites.

The effect of manure on the GI persisted over three years in the form of stimulation of this parameter. This effect was strongest in the second year and amounted to 96% and 66%. It was weaker in the third year and finally disappeared.

**Figure 8:**
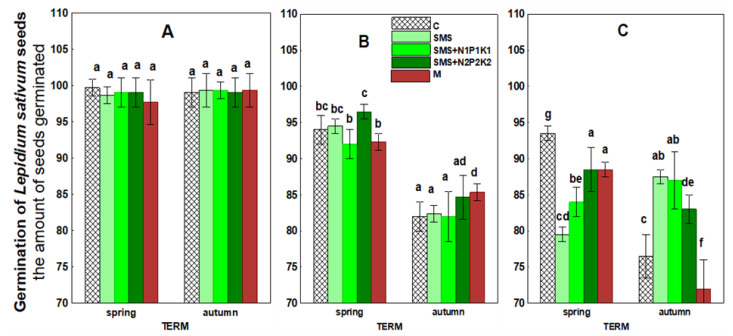
*Lepidium sativum* seed germination in the control soil and soil under different treatment strategies. (**A**)—1st year; (**B**)—2nd year; (**C**)—3rd year. C—control soil; SMS—soil + spent mushroom substrate; SMS + N1P1K1—soil + spent mushroom substrate + mineral fertilization N1P1K1; SMS + N2P2K2—soil + spent mushroom substrate + mineral fertilization N2P2K2; M—soil + manure. The vertical lines indicate the standard deviation. Different letters above the columns indicate significant differences at *p* < 0.05, and each year was analyzed independent of each other.

The results concerning seed germination of the test plant are shown in Figure 8 and Table 3. The application of spent mushroom substrate in different variants had no significant effect on this parameter during the first two years of the experiment. In the third year, the germination process differed between individual time points. In the spring, germination was inhibited in all sites with SMS. Inhibition became most apparent when spent mushroom waste was applied alone. The number of germinated seeds in this site was 79 compared to 93 in the control. In autumn, however, the process of seed germination was stimulated. The strongest increase in the number of germinated seeds was also observed in the site with waste alone and with waste applied in combination with N1P1K1, which amounted to 88 and 86 seeds, respectively, compared to 77 seeds in the control.

The effect of manure also occurred only with time and was not uniform. In the second year of the study, there was a significant stimulation of seed germination in the fall, with the number of seeds reaching 85 compared to 82 in the control. In contrast, the negative effect of manure on this process became apparent in the third year at both time points. There was also a decrease in the number of germinated seeds (from 94 and 77 in the control soil to 89 and 72 in the soil with manure). 

**Figure 9:**
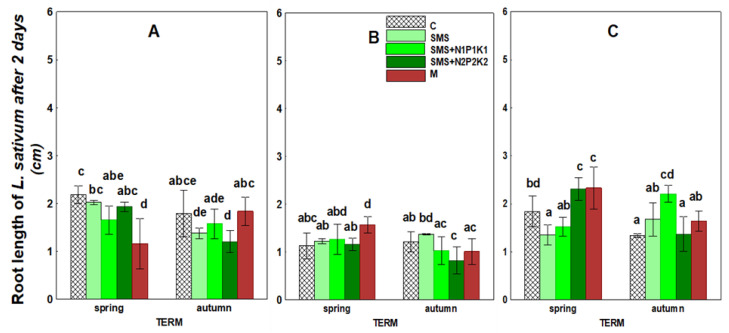
Increase in root length of *Lepidium sativum* in control soil and soil in different treatment strategies after two days. (**A**)—1st year; (**B**)—2nd year; (**C**)—3rd year. C—control soil; SMS—soil + spent mushroom substrate; SMS + N1P1K1—soil + spent mushroom substrate + mineral fertilization N1P1K1; SMS + N2P2K2—soil + spent mushroom substrate + mineral fertilization N2P2K2; M—soil + manure. The vertical lines indicate the standard deviation. Different letters above the columns indicate significant differences at *p* < 0.05, and each year was analyzed independent of each other.

**Figure 10:**
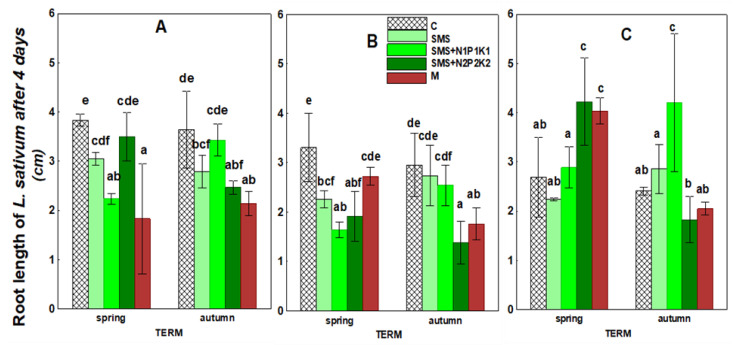
Increase in root length of *Lepidium sativum* in control soil and soil in different treatment strategies after four days. (**A**)—1st year; (**B**)—2nd year; (**C**)—3rd year. C—control soil; SMS—soil + spent mushroom substrate; SMS + N1P1K1—soil + spent mushroom substrate + mineral fertilization N1P1K1; SMS + N2P2K2—soil + spent mushroom substrate + mineral fertilization N2P2K2; M—soil + manure. The vertical lines indicate the standard deviation. Different letters above the columns indicate significant differences at *p* < 0.05, and each year was analyzed independent of each other.

The data shown in Figure 9 and Figure 10, as well as Table 3, refer to the increase in root length of the test plant measured after 2 and 4 days, respectively. The results showed that the applied waste and manure significantly affected the growth of the roots of the test plant seedlings. These changes developed with varying intensity over the three years of the study. However, in the case of root growth measured after four days, these alterations occurred in a greater number of sites. 

In the first year, there was a decrease in root growth (when compared to the control), both after two and four days. After two days, the lowest root growth that was recorded in the fall was at the site with SMS and N2P2K2, and after four days in the spring it was at the site with SMS and N1P1K1. In the second year of the study, the negative impact of SMS on root growth after two days almost disappeared and persisted only in the site with N2P2K2. However, the inhibition was still present when the measurement was taken after four days. It was particularly pronounced in the spring because it occurred in all sites with SMS, while in the autumn it was present only in the site with SMS and N2P2K2. In the third year of the study, the negative effect of spent mushroom substrate on root growth after 2 and 4 days disappeared and occurred only in a single site with SMS alone. In the other combinations and time points, the parameters studied were subject to stimulation.

The effect of manure on root length growth after two and four days also showed some dynamics. Initially, inhibition of these parameters relative to controls was observed in the first two years, particularly when measured after four days. In the third year, as in the case of the spent mushroom substrate, the parameter was stimulated in the spring (after 2 and 4 days), and no effect was noted in the fall. 

## 4. Discussion

**Figure 11:**
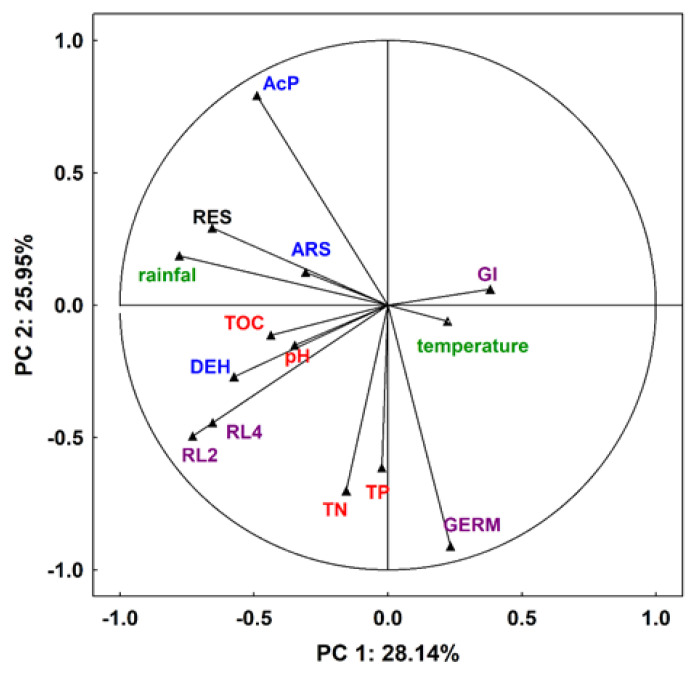
Principal component analysis (PCA) for the results of analyzed parameters in the soil-loading plot. RES—respiration of soil, DEH—dehydrogenases, ARS—arylsulfatase, AcP—acid phosphatase, GI—growth index of *L. sativum*, GERM—germination of *L. sativum*, RL2—root length of *L. sativum* after two days, RL4—root length of *L. sativum* after four days, TOC—total organic carbon, TN—total nitrogen, and TP—total potassium.

Soil respiration is a particularly important parameter in assessing the condition and quality of the soil, and thus the fertilizer value of various types of organic waste, since it reflects the full range of its biological activity. CO_2_ released in this process is derived mainly from the decomposition of organic matter by soil microorganisms (SOM) [31]. The rate of organic carbon mineralization depends on, among others, temperature, humidity, salinity, pH, and soil aeration, as these factors are closely related to the living conditions of soil microorganisms [54]. However, as the available literature shows, carbon mineralization is primarily related to organic matter [55]. Therefore, the stimulation—albeit with varying degrees of intensity—of respiratory activity observed in the current study was likely due to the input of organic matter along with spent mushroom substrate and manure, i.e., sources of respiratory substrates for soil microorganisms. Stimulation of respiratory processes by the addition of organic matter to the soil has also been reported by other authors [8,11,12,56]. The initial increase in respiratory activity in soil may have been due to the decomposition of readily available compounds brought in with spent mushroom substrate. On the other hand, the decrease in the activity of the analyzed parameter (which is noted later) could be the result of the depletion of these compounds or the induction phase of microbial enzymes. Additionally, it could also be the result of uptake and storage of carbon by microorganisms, without its utilization for cell structure repair or growth [57]. However, in the third year of the present study, significant stimulation of respiratory activity in all variants was observed, including the control soil, which could suggest that environmental conditions could have affected this analyzed parameter. This was confirmed by a cluster analysis which showed a positive correlation between respiration and precipitation and a negative correlation with temperature (Figure 11). 

**Figure 12:**
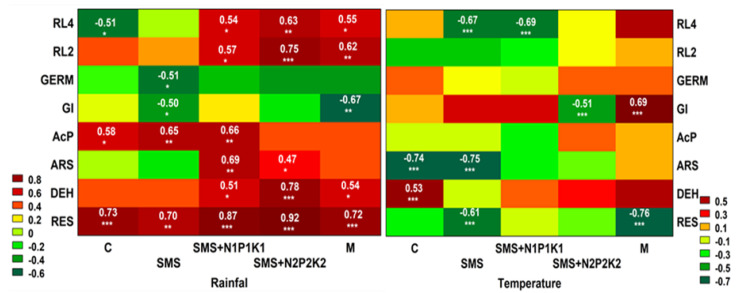
Heat map displaying the Pearson correlation coefficients between environmental factors (rainfall and temperature); biochemical and enzymatic activity; and phytotoxic parameters; as well as physicochemical and chemical properties at the combination level. Significance noted at * *p* < 0.05; ** *p* < 0.01; and *** *p* < 0.001, respectively. RL4—root length of *L. sativum* after four days, RL2—root length of *L. sativum* after two days, GERM—germination of *L. sativum*, GI—growth index of *L. sativum*, AcP—acid phosphatase, ARS—arylsulfatase, DEH—dehydrogenases, RES—respiration of soil, C—control soil; SMS—soil + spent mushroom substrate; SMS + N1P1K1—soil + spent mushroom substrate + mineral fertilization N1P1K1; SMS + N2P2K2—soil + spent mushroom substrate + mineral fertilization N2P2K2; M—soil + manure.

With regard to temperature, negative correlations were recorded only in the variants with SMS alone and manure (Figure 12). Perhaps the key role in the other variants was played by the applied supplemental mineral fertilization, which helped stimulate the decomposition of soil organic matter. The available literature shows that low doses of this type of fertilization have a beneficial effect on the microbiological and agrochemical properties of the soil, as they accelerate decomposition and increase the amount of soil organic matter [58,59,60]. Hernandez et al. [56] point out that combined organic and mineral fertilization is a good substitute for mineral nitrogen fertilization. For precipitation, positive correlations in all variants were recorded and, in combinations with different mineral fertilization variants, they were at a significance level of *p* < 0.001, similar to manure, while in the variant with SMS alone, they were at a fairly high level of *p* < 0.01 (Figure 12).

**Figure 13:**
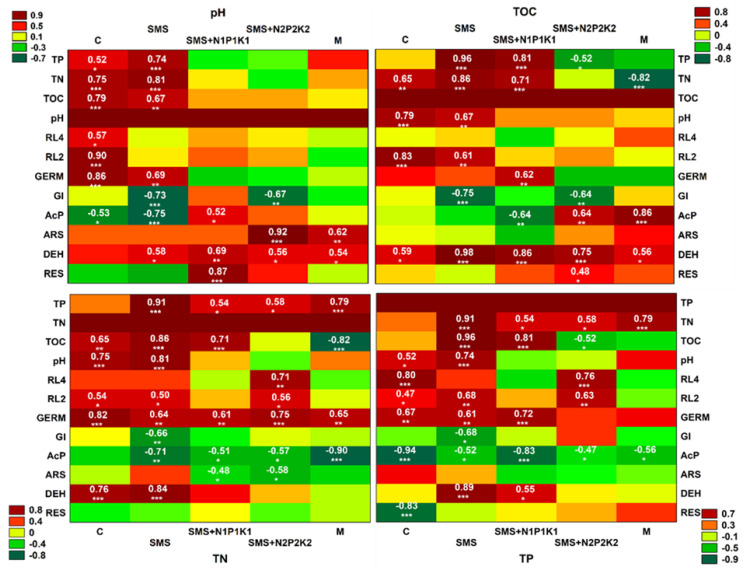
Heat map displaying the Pearson correlation coefficients between chemical and physico-chemical properties; biochemical and enzymatic activity; and phytotoxic parameters at the combination level. Significance noted at * *p* < 0.05; ** *p* < 0.01; and *** *p* < 0.001, respectively. TOC—total organic carbon, TN—total nitrogen, TP—total potassium, RL4—root length of *L. sativum* after four days, RL2—root length of *L. sativum* after two days, GERM—germination of *L. sativum*, GI—growth index of *L. sativum*, AcP—acid phosphatase, ARS—arylsulfatase, DEH—dehydrogenases, RES—respiration of soil, C—control soil; SMS—soil + spent mushroom substrate; SMS + N1P1K1—soil + spent mushroom substrate + mineral fertilization N1P1K1; SMS + N2P2K2—soil + spent mushroom substrate + mineral fertilization N2P2K2; M—soil + manure.

Reports of other authors have confirmed the current observations, as they have also recognized the relationship of soil fertilization with spent mushroom substrate and weather conditions [61]. This may be due to the ability of the spent mushroom substrate to retain water in the soil which, in turn, results in the better reaction of crops to periodic drought conditions. This is a compelling argument that can give SMS an advantage over other fertilizers in terms of its impact on yield and crop quality. Other physicochemical and chemical parameters did not play a significant role in the respiratory activity analyzed in the current study. Positive correlations were found with pH only in the case of variants with mineral fertilization at a significance level of *p* < 0.001 (SMMS+N1P1K1) and with TOC at *p* < 0.05 (SMS+N2P2K2) (Figure 13). The effects of organic waste on respiratory activity were also analyzed with varying results, in studies, among others, by Joniec [21], Álvarez-Martín et al. [32], Elsakhawy and El-Rahem [33], Joniec et al. [34], and Paula et al. [35].

Microbial respiration is also related to the activity of dehydrogenases, whose determination allows controlling changes in the population of soil microorganisms, which is an important parameter of soil quality. Soil enzymes are biological catalysts for many biochemical processes in the soil environment, including those related to the emission of greenhouse gases CO_2_ and N_2_O [62]. They are also suitable markers of soil fertility as they are involved in the cycle of the most important nutrients [63,64]. It is well known that dehydrogenase activity in soil depends on organic carbon content. Therefore, as in the case of biochemical activity, it can be assumed that these were the transformation products of spent mushroom substrate organic matter and the changes they induced in the soil environment that contributed to the stimulation of dehydrogenase activity in the current study. This was confirmed by a cluster analysis that showed positive correlations of the analyzed enzyme with TOC, TN, TP, and pH (Figure 11). At the same time, it should be noted that the correlations with TOC of the variants with SMS were at the significance level of *p* < 0.001, and for manure only at *p* < 0.05 (Figure 13). As in the case of respiration, the initial increase in dehydrogenase activity was probably caused by the introduction of readily degradable nutrients into the soil along with SMS, which resulted in improved conditions for many microbial groups, and this translated into the stimulation of dehydrogenases. The improvement of these conditions was also evidenced by the recorded significant correlations of the enzymes with pH in all variants with SMS at *p* < 0.01 (SMS + N1P1K1) and *p* < 0.05 (SMS and SMS + N2P2K2). For manure, the recorded significance of the results was also at the level of *p* < 0.05 (Figure 13). In contrast, the later decrease in the activity of these enzymes was probably caused by the breakdown of more readily available nutrients. The dynamics of changes in the activity of dehydrogenases, similarly to biochemical activity, could also have been caused by environmental conditions, as evidenced by positive correlations with precipitation and temperature (Figure 11 and Figure 12). Dehydrogenases are fairly sensitive to changes associated with seasons because they are closely associated with the dynamics of microbial activity [65]. The effect of spent mushroom substrate medium on dehydrogenase activity was studied by, among others, Meng et al. [16], Álvarez-Martín et al. [32], Elsakhawy and El-Rahem [33], and Gong et al. [66].

Other enzymes involved in the circulation of major nutrients are acid phosphatase and arylsulfatase, which are associated with phosphorus and sulfur metabolism. They catalyze transformations of various substrates, releasing available inorganic forms of phosphate and sulfate, which serve as key energy sources for plants and soil organisms. They are also sensitive indicators of agriculture-induced changes in soil properties due to their strong association with soil organic matter content and quality [67,68]. Cluster analysis showed a positive correlation of acid phosphatase and arylsulfatase with TOC, but in the combined variants, significant results were recorded only for phosphatase (Figure 11 and Figure 13). In addition, the activity of the discussed enzymes was generally inhibited by the wastes applied, although their negative effect weakened over time and even disappeared; however, for arylsulfatase, it persisted even in the third year (SMS+N1P1K1). Therefore, we could surmise that organic matter introduced in the form of spent mushroom substrate and manure did not play a key role in the activity of these enzymes. Hence, it disproves the first hypothesis regarding the fertilizing qualities of the spent mushroom substrate. As demonstrated by other authors, phosphatase activity may inhibit the presence of mineral phosphorus in the soil [68,69,70]. It was likely that this factor also played a key role in the present study, as evidenced by the recorded negative correlations between phosphatase activity and the content of bioavailable mineral phosphorus (Figure 11 and Figure 13). TN was another parameter that could influence the activity of the discussed enzymes. Cluster analysis showed negative correlations of this factor with both acid phosphatase and arylsulfatase (Figure 11). For the first enzyme, negative correlations with TN were recorded in all variants with the spent mushroom substrate, while with SMS alone, they were at *p* < 0.01, and with mineral fertilization they were at *p* < 0.05 (Figure 13). For arylsulfatase, negative correlations at the *p* < 0.05 level were recorded only in combinations with mineral fertilization (Figure 13). Likely it was the addition of nitrogen in the form of mineral fertilization that increased the availability of sulfur in the soil, and this translated into a decrease in the activity of arylsulfatase. Similar conclusions were reached, among others, by Mori et al. [71], while Sawicka et al. [72] noted a significant effect of mineral fertilization on the activity of acid phosphatase. Another possible cause for alterations in the activity of the analyzed hydrolases was the change in the soil pH. This assumption was confirmed by the observed positive correlations between the analyzed enzymes, but it was much stronger in the case of arylsulfatase (Figure 11 and Figure 13); these differences were probably due to the various sensitivity of these enzymes to this same chemical parameter [68]. The activity of these enzymes could also be influenced by environmental conditions, as evidenced by positive correlations with rainfall and negative correlations with temperature (Figure 11 and Figure 12). Both hydrolases are frequently utilized to assess the condition of soil environments, including those fertilized with various types of organic waste [21,34,41,42,43,73].

Spent mushroom substrate introduced into soil generally has a positive effect on the physical, chemical, and microbiological properties of the soil environment. However, the introduction of waste organic matter into the soil also carries a certain risk of disturbing the living conditions of plants. Therefore, it is important to monitor the effects of unconventional organic fertilizers, such as spent mushroom substrate, on parameters related to plant growth and development. The conducted research shows that the organic matter and mineral compounds that were introduced with spent mushroom substrate and manure contributed to the stimulation of *L. sativum* growth in the initial period. This was likely caused by better availability of valuable nutrients, important from the point of view of plant nutrition, and a better aggregate structure of the soil. On the other hand, the decrease in the activity of this parameter observed later could be related to the activation of previously unavailable pollutants as a result of organic matter mineralization. Joniec et al. [21] reached similar conclusions in their study. Transformations of organic carbon in the soil influenced not only GI, but also the germination of *L. sativum*, as evidenced by the observed negative correlations with soil respiration and in the case of GI with dehydrogenase (Figure 11). In turn, with regard to the increment in the root length, cluster analysis showed positive correlations of this parameter with both biochemical and enzymatic activity, but only after two days (Figure 11). The ecotoxicological parameters related to plant growth were also likely influenced by the transformation of other nutrients, which was confirmed by the reported negative correlations of GI with arylsulfatase and in the germination with acid phosphatase (Figure 11). Chemical parameters such as TN and TP also played an important role, especially with respect to germination and root growth. This was confirmed by significantly positive correlations of germination with TN for all the experimental variants at the significance level of *p* < 0.001 and *p* < 0.01 and for the root growth (after two days) at the level of *p* < 0.05 (Figure 13). TP also played an important role in these parameters, as demonstrated by correlations at the level of *p* < 0.001 and *p* < 0.01 for virtually all combinations (Figure 13). The influence of these elements on the growth of *L. sativum* was also reported by Mohamed et al. [74]. The results of the current study concerning *L. sativum* root length, measured after two and four days, indicated that this parameter was most sensitive to potentially harmful compounds occurring or resulting from changes in organic matter introduced with SMS and M in soil solution. Similar conclusions were also reached by Godlewska et al. [20]. The influence of the tested fertilizing materials on this parameter was fairly varied and depended on the combination and duration of the experiment. The decrease in toxicity, in this case, could probably be related to a reduced effect of the toxic agent as a result of its degradation or leaching. The analyzed parameters related to phytotoxicity may also be influenced by SMS composition because, as reported by Catal and Peksen [73], ammonia, salts, various heavy metals, or low molecular weight organic compounds present in SMS may also prevent seed germination and root development. The data presented in Table 1 indicate that a certain pool of heavy metals was brought in together with the spent mushroom substrate each year in the discussed experiment. The obtained results showed that the overall physicochemical and chemical conditions in the analyzed soil after the addition of spent mushroom substrate positively influenced the initial development of the plants. The adverse effects of SMS on the studied parameters were most apparent in soil solutions, which would indicate that improving the aforementioned soil conditions eliminated the negative impact of compounds present in the soil solution. On the other hand, Canellas and Olivare [75] reported that plants grown under optimal nutritional conditions spent less energy on growing roots. The results obtained in this study, therefore, are difficult to relate to the data of other authors due to the scarcity of reports concerning the effect of spent mushroom substrate on such parameters as the growth index, germination, and root length increment in L. *sativum* [73], strictly speaking.

## 5. Conclusions

The use of spent mushroom substrate significantly increased the parameters related to microbiological soil carbon transformations, i.e., respiration and dehydrogenase activity. The intensity of the respiration process, measured by the amount of CO_2_ and the activity of dehydrogenases, was maintained with varying intensity throughout the research period in sites where waste was applied jointly with mineral fertilization. For respiration, the highest CO_2_ release was recorded in the third year of the study. These observations indicate that waste matter has been incorporated into the microbial processes involved in the carbon cycle. This study partially confirms that spent mushroom substrate is a good fertilizer for increasing soil microbial activity and that it can be applied every year. On the other hand, the activity of enzymes responsible for phosphorus and sulfur metabolism, i.e., phosphatase and arylsulfatase, was inhibited by a spent mushroom substrate. It should be noted that the negative effect of waste weakened and even disappeared over time, but it persisted in the case of arylsulfatase also in the third year in the sites with waste and N1P1K1 fertilization. Therefore, the hypothesis concerning the fertilization values of a spent mushroom substrate, which can be applied every year, was rejected, in part, concerning its influence on the transformation of phosphorus, especially sulfur.

The obtained results showed that the fertilizing application of spent mushroom substrate contributed to an increase in the amount of released CO_2_, which increased over time. Unfortunately, these observations do not confirm that such a method of waste management does not contribute to the exacerbation of the greenhouse effect by increasing CO_2_ emissions from the soil. In this context, manure proved to be a safer fertilizer as it did not cause a significant persistent increase in CO_2_ emissions.

The results of the effect of spent mushroom substrate on parameters related to the initial stage of the test plant development showed that its nature varied depending on the time period. A positive effect was noted for GI and root growth, but only in the third year of the study. In the initial year, root growth was lower in the sites with a spent mushroom substrate. Similar observations apply to the impact of manure. The results of the research on the impact of a spent mushroom substrate on phytotoxicity confirmed that the growth index was not negatively affected. On the other hand, considering the root growth, whose inhibition was recorded in the first and second years of the study, it should be concluded that soil phytotoxicity deteriorated periodically.

It should be emphasized that one of the goals of sustainable development is to preserve or increase soil fertility, while also reducing GHG emissions from the agricultural sector. The obtained results may be helpful in making decisions on the fertilization of mushroom waste in light of the principles of sustainable development.

## Figures and Tables

**Table 1:** Properties of soil and wastes [44].

Property	Unit	Soil	Spent Mushroom Substrate	Manure
pH_KCl_	1 mol KCl	7.0	6.6	7.3
TOC	g kg^−1^	14.98	105.0	135.8
TN	g kg^−1^	1.51	6.50	9.47
TP	g kg^−1^	0.19	0.25	0.25
Ca	mg kg^−1^	1660	15,800	2240
K		2350	6330	11,100
Mg		1390	1240	1550
Zn	mg kg^−1^	No.	86.0	No.
Cu			16.6	
Ni			2.81	
Cr			1.84	
Cd			0.055	
Pb			0.956	
Hg			0.07	

Abbreviations: TOC—total organic carbon, TN—total nitrogen, and TP—total potassium.

**Table 2:** Selected physico-chemical and chemical properties of the soil [44].

	Year	Season	C	SMS	SMS + N1P1K1	SMS + N2P2K2	M
pH 1 mol KCl	2018	spring	7.03	7.20	6.41	5.16	7.47
		autumn	6.86	7.60	5.98	6.60	5.44
	2019	spring	6.42	6.75	5.88	5.84	6.20
		autumn	6.34	6.04	6.18	5.53	6.24
	2020	spring	6.87	6.85	6.68	6.79	6.56
		autumn	6.25	6.13	6.33	6.64	6.50
TOC g kg^−1^	2018	spring	14.98	19.50	17.21	12.83	13.45
		autumn	13.59	14.39	14.34	11.46	12.16
	2019	spring	12.19	12.99	14.75	15.60	14.89
		autumn	12.02	10.63	13.25	13.28	18.18
	2020	spring	15.62	16.30	14.90	15.33	17.75
		autumn	13.34	12.54	13.85	14.91	14.78
TN g kg^−1^	2018	spring	1.51	1.82	2.13	1.46	1.36
		autumn	1.37	1.44	1.39	1.18	1.28
	2019	spring	1.50	1.10	1.00	1.30	1.10
		autumn	0.96	0.97	1.30	0.84	1.00
	2020	spring	1.70	1.20	0.98	1.40	1.10
		autumn	0.97	0.80	1.20	0.55	1.10
TP g kg^−1^	2018	spring	0.19	0.21	0.21	0.17	0.22
		autumn	0.16	0.16	0.14	0.15	0.18
	2019	spring	0.15	0.13	0.19	0.10	0.10
		autumn	0.11	0.10	0.11	0.13	0.15
	2020	spring	0.10	0.15	0.12	0.16	0.15
		autumn	0.12	0.13	0.14	0.11	0.14

Abbreviations: TOC—total organic carbon, TN—total nitrogen, and TP—total potassium. C—control soil, SMS—soil + spent mushroom substrate, SMS + N1P1K1—soil + spent mushroom substrate + mineral fertilization N1P1K1, SMS + N2P2K2—soil + spent mushroom substrate + mineral fertilization N2P2K2, and M—soil + manure.

**Table 3:** Biochemical, enzymatic activity and soil phytotoxicity parameters (annual averages).

Years	Experimental Treatments	RES	DEH	AcP	ARS	GI	GERM	RL2	RL4
2018	C	67.28 a	4.89 abc	36.60 b	63.95 i	100.00 ab	99.33 g	1.99 f	3.74 g
	SMS	80.58 cd	11.60 i	38.58 b	60.43 h	92.86 a	99.00 g	1.70 de	2.92 de
	SMS + N1P1K1	143.84 h	9.23 gh	34.25 b	27.44 c	93.75 a	99.17 g	1.62 de	2.83 cde
	SMS + N2P2K2	111.40 f	3.77 a	24.35 a	23.58 a	176.86 e	99.00 g	1.57 d	2.98 def
	M	89.65 d	4.88 ab	23.60 a	31.28 d	145.86 d	98.50 g	1.50 cd	1.99 ab
2019	C	80.75 cd	5.31 bc	48.76 c	42.58 ef	100.00 ab	88.00 de	1.18 ab	3.13 efg
	SMS	77.76 bc	3.94 a	48.93 c	45.51 g	220.27 f	88.42 ef	1.30 bc	2.50 bcd
	SMS + N1P1K1	101.64 e	4.64 ab	43.92 c	26.50 bc	144.20 d	87.00 cde	1.15 ab	2.09 ab
	SMS + N2P2K2	100.92 e	4.37 ab	57.06 de	24.65 ab	116.94 abc	90.58 f	1.00 a	1.65 a
	M	70.38 ab	6.01 cd	55.59 d	40.23 e	181.52 e	88.83 ef	1.30 bc	2.24 abc
2020	C	121.85 g	6.72 de	61.98 ef	46.72 g	100.00 ab	85.00 bc	1.59 d	2.55 bcde
	SMS	121.63 g	7.80 ef	62.14 ef	42.06 ef	134.31 cd	83.50 b	1.52 cd	2.55 bcde
	SMS + N1P1K1	234.88 i	9.66 h	67.60 g	41.06 e	145.80 d	85.50 bc	1.87 ef	3.55 fg
	SMS + N2P2K2	237.80 i	8.77 fgh	65.00 fg	44.56 fg	92.83 a	85.75 bcd	1.84 ef	3.03 def
	M	116.22 fg	8.25 fg	64.69 fg	46.56 g	122.13 bcd	80.25 a	1.99 f	3.04 def

Abbreviations: C—control soil; SMS—soil + spent mushroom substrate; SMS + N1P1K1 soil + spent mushroom substrate + mineral fertilization N1P1K1; SMS + N2P2K2—soil + spent mushroom substrate + mineral fertilization N2P2K2; M—soil + manure; RES—respiration of soil (mg CO_2_ kg^−1^ d.m. of soil d^−1^); DEH—dehydrogenases (mg TPF kg^−1^ d.m. of soil d^−1^); AcP—acid phosphatase (mg PNP kg^−1^ d.m. of soil h^−1^); ARS—arylsulfatase (mg PNP kg^−1^ d.m. of soil h^−1^); GI—growth index *L. sativum* (%); GERM—germination of *L. sativum* (the number of seeds germinated); RL2—root length of *L. sativum* after two days (cm); and RL4—root length of *L. sativum* after four days (cm). Different letters indicate significant differences at *p* < 0.05.

## Data Availability

Additional information can be provided by the corresponding author.

## References

[1] Adejumo I.O., Adebiyi O.A., Saleh H.M. (2020). Agricultural solid wastes: Causes, effects, and effective management. Strategies of Sustainable Solid Waste Management.

[2] Hanafi M.F.H., Rezania S., Taib M.S., Md Din M.F., Yamauchi M., Sakamoto M., Hara H., Park J., Ebrahimi S.S. (2018). Environmentally sustainable applications of agro-based spent mushroom substrate (SMS): An overview. J. Mater. Cycles Waste Manag..

[3] FAOSTAT (2022). Crops and Livestock Products. https://www.fao.org/faostat/en/#rankings/countries_by_commodity.

[4] Cunha Zied D., Sánchez J.E., Noble R., Pardo-Giménez A. (2020). Use of spent mushroom substrate in new mushroom crops to promote the transition towards a circular economy. Agronomy.

[5] Leong Y.K., Ma T.W., Chang J.S., Yang F.C. (2022). Recent advances and future directions on the valorization of spent mushroom substrate (SMS): A review. Bioresour. Technol..

[6] Rinker D.L., Cunha Zied D., Pardo-Gimenez A. (2017). Spent mushroom substrate uses. Edible and Medicinal Mushrooms: Technology and Applications.

[7] Kwiatkowski C.A., Harasim E. (2021). The Effect of Fertilization with Spent Mushroom Substrate and Traditional Methods of Fertilization of Common Thyme (*Thymus vulgaris* L.) on Yield Quality and Antioxidant Properties of Herbal Material. Agronomy.

[8] Owaid M.N., Abed I.A., Al-Saeedi S.S.S. (2017). Applicable properties of the bio-fertilizer spent mushroom substrate in organic systems as a byproduct from the cultivation of *Pleurotus* spp.. Inform. Process. Agric..

[9] Prasad R., Lisiecka J., Kleiber T. (2022). Morphological and Yield Parameters, Dry Matter Distribution, Nutrients Uptake, and Distribution in Strawberry (*Fragaria* × *ananassa* Duch.) cv. ‘Elsanta’ as Influenced by Spent Mushroom Substrates and Planting Seasons. Agronomy.

[10] Velusami B., Jordan S.N., Curran T., Grogan H. (2021). Fertiliser characteristics of stored spent mushroom substrate as a sustainable source of nutrients and organic matter for tillage, grassland and agricultural soils. Irish, J. Agric. Food Res..

[11] Frąc M., Pertile G., Panek J., Gryta A., Oszust K., Lipiec J., Usowicz B. (2021). Mycobiome composition and diversity under the long-term application of spent mushroom substrate and chicken manure. Agronomy.

[12] Zhou H., Fang H., Zhang Q., Wang Q., Chen C., Mooney S.J., Peng X., Du Z. (2019). Biochar enhances soil hydraulic function but not soil aggregation in a sandy loam. Eur. J. Soil Sci..

[13] Lipiec J., Usowicz B., Kłopotek J., Turski M., Frąc M. (2021). Effects of Application of Recycled Chicken Manure and Spent Mushroom Substrate on Organic Matter, Acidity, and Hydraulic Properties of Sandy Soils. Materials.

[14] Malińska K., Czekała W., Janczak D., Dach J., Mazurkiewicz J., Dróżdż D. (2018). Spent mushroom substrate as a supplementary material for sewage sludge composting mixtures. Environ. Prot. Eng..

[15] Grimm D., Wösten H.A.B. (2018). Mushroom cultivation in the circular economy. Appl. Microbiol. Biotechnol..

[16] Meng L., Zhang S., Gong H., Zhang X., Wu C., Li W. (2018). Improving sewage sludge composting by addition of spent mushroom substrate and sucrose. Bioresour. Technol..

[17] Kucaj W.F., Rygielski K., Cybulska K. (2019). Optimizing the use of the phytotoxkit test to assess the toxicity of soil contaminated with creosote. Pol. J. Soil Sci..

[18] Szymanski M., Dobrucka R. (2022). Application of Phytotests to Study of Environmental Safety of Biologically Synthetised Au and Au/ZnO Nanoparticles Using *Tanacetum parthenium* Extract. J. Inorg. Organomet. Polym. Mater..

[19] Alvarenga P., Mourinha C., Farto M., Santos T., Palma P., Sengo J., Morais M.-C., Cunda-Queda C. (2015). Sewage sludge, compost and other representative organic wastes as agricultural soil amendments: Benefits versus limiting factors. Waste Manag..

[20] Godlewska P., Jośko I., Oleszczuk P. (2022). Ecotoxicity of sewage sludge- or sewage sludge/willow-derived biochar-amended soil. Environ. Pollut..

[21] Joniec J., Oleszczuk P., Jezierska-Tys S., Kwiatkowska E. (2019). Effect of reclamation treatments on microbial activity and phytotoxicity of soil degraded by the sulphur mining industry. Environ Pollut..

[22] Manas P., De las Heras J. (2018). Phytotoxicity test applied to sewage sludge using *Lactuca sativa* L. and *Lepidium sativum* L. seeds. Int. J. Environ. Sci. Technol..

[23] Pampuro N., Bisaglia C., Romano E., Brambilla M., Foppa Pedretti E., Cavallo E. (2017). Phytotoxicity and Chemical Characterization of Compost Derived from Pig Slurry Solid Fraction for Organic Pellet Production. Agriculture.

[24] Allen J., Pascual K.S., Romasanta R.R., Van Trinh M., Van Thach T., Van Hung N., Chivenge P., Gummert M., Hung N., Chivenge P., Douthwaite B. (2020). Rice straw management effects on greenhouse gas emissions and mitigation options. Sustainable Rice Straw Management.

[25] Rahman M.M. (2013). Carbon dioxide emission from soil. Agric. Res..

[26] Minasny B., Malone B.P., McBratney A.B., Angers D.A., Arrouays D., Chambers A., Chaplot V., Chen Z.S., Cheng K., Das B.S. (2017). Soil carbon 4 per mille. Geoderma.

[27] Navarro-Pedreño J., Almendro-Candel M.B., Zorpas A.A. (2021). The Increase of Soil Organic Matter Reduces Global Warming, Myth or Reality?. Science.

[28] Bond-Lamberty B., Thomson A. (2010). Temperature-associated increases in the global soil respiration record. Nature.

[29] Le Quéré C., Andrew R.M., Friedlingstein P., Sitch S., Hauck J., Pongratz J., Pickers P.A., Korsbakken J.I., Peters G.P., Canadell J.G. (2013). Global carbon budget. Earth Syst. Sci. Data.

[30] Joshi Gyawali A., Lester B.J., Stewart R.D. (2019). Talking SMAAC: A new tool to measure soil respiration and microbial activity. Front. Earth Sci..

[31] Kuzyakov Y. (2006). Sources of CO_2_ efflux from soil and review of partitioning methods. Soil Biol. Biochem..

[32] Álvarez-Martín A., Hilton S.L., Bending G.D., Rodríguez-Cruz M.S., Sánchez-Martín M.J. (2016). Changes in activity and structure of the soil microbial community after application of azoxystrobin or pirimicarb and an organic amendment to an agricultural soil. App. Soil Ecol..

[33] Elsakhawy T.A., El-Rahem W.T.A. (2020). Evaluation of Spent Mushroom Substrate Extract as a Biofertilizer for Growth Improvement of Rice (*Oryza sativa* L). Egypt. J. Soil Sci..

[34] Joniec J., Żukowska G., Bik-Małodzińska M., Kwiatkowska E., Rojek K. (2021). Reaction of Microorganisms to Long-Term Waste Reclamation of Soil Degraded by the Sulfur Mining Industry. Minerals.

[35] Paula F.S., Tatti E., Abram F., Wilson J., O’Flaherty V. (2017). Stabilisation of spent mushroom substrate for application as a plant growth-promoting organic amendment. J. Environ. Manag..

[36] Fanin N., Mooshammer M., Sauvadet M., Meng C., Alvarez G., Bernard L., Bertrand I., Blagodatskaya E., Bon L., Fontaine S. (2022). Soil enzymes in response to climate warming: Mechanisms and feedbacks. Funct. Ecol..

[37] Zi H.B., Hu L., Wang C., Wang G., Wu P., Lerdau M., Ade L. (2018). Responses of soil bacterial community and enzyme activity to experimental warming of an alpine meadow. Eur. J. Soil Sci..

[38] Meng C., Tian D.S., Zeng H., Li Z.L., Chen H.Y.H., Niu S.L. (2020). Global meta-analysis on the responses of soil extracellular enzyme activities to Warming. Sci. Total Environ..

[39] Shi L., Guo Y., Ning J., Lou S., Hou F. (2020). Herbicide applications increase greenhouse gas emissions of alfalfa pasture in the inland arid region of northwest China. PeerJ.

[40] Song Y.Y., Jiang L., Song C.C., Wang X.W., Ma X.Y., Zhang H., Tan W.W., Gao J.L., Hou A.X. (2021). Microbial abundance and enzymatic activity from tussock and shrub soil in permafrost peatland after 6-year warming. Ecol. Indic..

[41] Alvarenga P., Rodrigues D., Mourinha C., Palma P., de Varennes A., Cruz N., Tarelho L.A.C., Rodrigues S. (2019). Use of wastes from the pulp and paper industry for the remediation of soils degraded by mining activities: Chemical, biochemical and ecotoxicological effects. Sci. Total Environ..

[42] Cheviron N., Amadou I., Grondin V., Marrauld C., Mougin C., Morvan T. (2021). Soil enzymatic activity data over eight years at the EFELE site, a long-term field experiment on effects of organic waste products and tillage practices. Data Brief..

[43] Joniec J. (2018). Enzymatic activity as an indicator of regeneration processes in degraded soil reclaimed with various types of waste. Int. J. Environ. Sci. Technol..

[44] Joniec J., Kwiatkowska E., Kwiatkowski C.A. (2022). Assessment of the Effects of Soil Fertilization with Spent Mushroom Substrate in the Context of Microbial Nitrogen Transformations and the Potential Risk of Exacerbating the Greenhouse Effect. Agriculture.

[45] Lal R., Bouma J., Brevik E., Dawson L., Field D.J., Glaser B., Hatano R., Hartemink A.E., Kosaki T., Lascelles B. (2021). Soils and Sustainable Development Goals of the United Nations: An International Union of Soil Sciences Perspective. Geoderma Reg..

[46] Sustainable Development Goals. https://www.un.org/sustainabledevelopment/sustainable-development-goals/.

[47] Polish Society of Soil Science (2009). Particle size distribution and textural classes of soils and mineral materials—Classification of Polish Society of Soil Science 2008. Soil Sci. Ann..

[48] WRB IUSS Working Group (2015). World Reference Base for Soil Resources 2014. update 2015; International soil classification system for naming soils and creating legends for soil maps. World Soil Resources Reports.

[49] Rühling A., Tyler G. (1973). Heavy metal pollutions and decomposition of Spruce Needle litter. Oikos.

[50] Thalmann A. (1968). Zur Methodik der Bestimmung der dehydrogenaseactivität im boden mittels triphenyltetrazoliumchlorid (TTC). Landwirtsch. Forsch..

[51] Tabatabai M.A., Bremner J.M. (1969). Use of p-nitrophenyl phosphate for assay of soil phosphatase activity. Soil Biol. Biochem..

[52] Tabatabai M.A., Bremner J.M. (1970). Arylosulfatase activity of soils. Soil Sci. Soc. Am. J..

[53] Masciandaro G., Ceccanti B., Garcia C. (1997). Soil agro-ecological management: Fertirrigation and vermicompost treatments. Bioresour. Technol..

[54] Grzyb A., Wolna-Maruwka A., Niewiadomska A. (2020). Environmental Factors Affecting the Mineralization of Crop Residues. Agronomy.

[55] Dai S., Hower J.C., Finkelman R.B., Graham I.T., French D., Ward C.R., Eskenzay G., Wei Q., Zhao L. (2020). Organic associations of non-mineral elements in coal: A review. Int. J. Coal. Geol..

[56] Hernandez T., Berlanga J.G., Tormos I., Garcia C. (2021). Organic versus Inorganic Fertilizers: Response of Soil Properties and Crop Yield. AIMS Geosci..

[57] Medina E., Paredes C., Bustamante M.A., Moral R., Moreno-Caselles J. (2012). Relationships between soil physico-chemical, chemical and biological properties in a soil amended with spent mushroom substrate. Geoderma.

[58] Chen J., Song D., Liu D., Sun J., Wang X., Zhou W., Liang G. (2022). Soil Aggregation Shaped the Distribution and Interaction of Bacterial-Fungal Community Based on a 38-Year Fertilization Experiment in China. Front Microbiol..

[59] Kátai J., Zsuposné A.O., Tállai M., Alshaal T. (2020). Would fertilization history render the soil microbial communities and their activities more resistant to rainfall fluctuations?. Ecotoxicol. Environ. Saf..

[60] Sivojiene D., Kacergius A., Baksiene E., Maseviciene A., Zickiene L. (2021). The influence of organic fertilizers on the abundance of soil microorganism communities, agrochemical indicators, and yield in east lithuanian light soils. Plants.

[61] Guo M., Chorover J. (2004). Solute release from weathering of spent mushroom substrate under controlled conditions. Compost. Sci. Util..

[62] Wojewódzki P., Lemanowicz J., Debska B., Haddad S.A. (2022). Soil Enzyme Activity Response under the Amendment of Different Types of Biochar. Agronomy.

[63] Gianfreda L., Rao M.A. (2014). Enzymes in Agricultural Sciences.

[64] Hu H., Zhou H., Zhou S., Li Z., Wei C., Yu Y., Hay A.G. (2019). Fomesafen impacts bacterial communities and enzyme activities in the rhizosphere. Environ. Pollut..

[65] Wolińska A., Stępniewska Z., Canuto R.A. (2012). Dehydrogenase Activity in the Soil Environment. Dehydrogenases.

[66] Gong X., Li S., Carson M.A., Chang S.X., Wu Q., Wang L., An Z., Sun X. (2019). Spent mushroom substrate and cattle manure amendments enhance the transformation of garden waste into vermicomposts using the earthworm *Eisenia fetida*. J. Environ. Manag..

[67] Acosta-Martinez V., Cano A., Johnson J. (2018). Simultaneous determination of multiple soil enzyme activities for soil health-biogeochemical indices. Appl. Soil Ecol..

[68] Dotaniya M.L., Aparna K., Dotaniya C.K., Singh M., Regar K.L., Kudus M. (2019). Role of soil enzymes in sustainable crop production. Enzymes in Food Biotechnology: Production, Applications, and Future Prospects.

[69] Manzoor A., Dippold M.A., Loeppmann S., Blagodatskaya E. (2022). Two-Phase Conceptual Framework of Phosphatase Activity and Phosphorus Bioavailability. Front. Plant. Sci..

[70] Perez-de-Mora A., Burgos P., Cabrera F., Madejon E., Trasar-Cepeda C., Hernandez T., Garcia C., Gonzalez-Carcedo S. (2012). Progress in microbial activity and chemical properties of a trace element polluted soil dunder assisted natural remediation. Soil Enzymology in the Recycling of Organic Wastes and Environmental Restoration.

[71] Mori T., Zhou K., Wang C., Wang S., Wang Y., Zheng M., Lu X., Zhang W., Mo J. (2020). Effects of 14-year continuous nitrogen addition on soil arylsulphatase and phosphodiesterase activities in a mature tropical forest. Glob. Ecol. Conserv..

[72] Sawicka B., Krochmal-Marczak B., Pszczółkowski P., Bielińska E.J., Wójcikowska-Kapusta A., Barbaś P., Skiba D. (2020). Effect of Differentiated Nitrogen Fertilization on the Enzymatic Activity of the Soil for Sweet Potato (*Ipomoea batatas* L. [Lam.]) Cultivation. Agronomy.

[73] Catal S., Peksen A. (2020). Physical, chemical and biological properties of spent mushroom substrates of different mushroom species. Acta Hortic..

[74] Mohamed E.A.A., Muddathir A.M., Abdalla A.H. (2020). Effects of organic and inorganic fertilization on growth, yield, seed fixed oil content, and fatty acids profile of garden cress (*Lepidium sativum* L.). SN Appl. Sci..

[75] Canellas L.P., Olivares F.L. (2014). Physiological responses to humic substances as a plant growth promoter. Chem. Biol. Technol. Agric..

